# Multidimensional Approach to Frailty

**DOI:** 10.3389/fpsyg.2020.00564

**Published:** 2020-03-25

**Authors:** Marta Wleklik, Izabella Uchmanowicz, Ewa A. Jankowska, Cristiana Vitale, Magdalena Lisiak, Marcin Drozd, Piotr Pobrotyn, Michał Tkaczyszyn, Christopher Lee

**Affiliations:** ^1^Faculty of Health Sciences, Wrocław Medical University, Wrocław, Poland; ^2^Centre for Heart Diseases, Faculty of Health Sciences, Wrocław Medical University, Wrocław, Poland; ^3^Centre for Clinical and Basic Research, IRCCS San Raffaele Pisana, Rome, Italy; ^4^Clinical University Hospital, Wrocław, Poland; ^5^William F. Conell School of Nursing, Boston College, Newton, MA, United States

**Keywords:** cardiology, determinants, disability, elderly, frailty syndrome, multi-morbidity

## Abstract

The concept of frailty syndrome (FS) was first described in the scientific literature three decades ago. For a very long time, we understood it as a geriatric problem, recently becoming one of the dominant concepts in cardiology. It identifies symptoms of FS in one in 10 elderly people. It is estimated that in Europe, 17% of elderly people have FS. The changes in FS resemble and often overlap with changes associated with the physiological aging process of the body. Although there are numerous scientific reports confirming that FS is age correlated, it is not an unavoidable part of the aging process and does not apply only to the elderly. FS is a reversible clinical condition. To maximize benefits of frailty-reversing activities for patient with frailty, identification of its determinants appears to be fundamental. Many of the determinants of the FS have already been known: reduction in physical activity, malnutrition, sarcopenia, polypharmacy, depressive symptom, cognitive disorders, and lack of social support. This review shows that insight into FS determinants is the starting point for building both the comprehensive definition of FS and the adoption of the assessment method of FS, and then successful clinical management.

## Introduction

There are an increasing number of research reports on frailty syndrome (FS) showing its importance in cardiology and evidence-base clinical practice. Guidelines for clinical management in cardiology emphasize the need to monitor FS and search for its reversible causes in the elderly (Ponikowski et al., 2016). Despite the widespread importance of FS in clinical management, there are no explicit cardiological guidelines adopting a specific definition of FS and requirements for applying methods of its identification (Vogt et al., 2018). In cardiology, there are no standardized methods in clinical decisions-making based on FS, as it is still being diagnosed with the patient’s foot-of-the-bed assessment or the so-called “eyeball test” (Bridgman et al., 2015). The Task Force of the International Conference of Frailty and Sarcopenia Research (ICFSR) has developed clinical practice guidelines for identification and management of physical frailty. These recommendations recognize that older adults over age 65 should be screened for FS rapidly based on the validated instrument adapted for the specific patient’s conditions. All patients who passed a positive screening test for frailty and patients classified as pre-frail should receive further assessments for clinical frailty (Dent et al., 2019).

According to the phenotypic approach, older adults are diagnosed as pre-frail when there are one or two components: weakness, slowness, weight loss, low physical activity, or exhaustion. Frailty is a dynamic condition, whereby pre-frail symptoms may develop into a full-blown frailty with the presence of three or more components, but may also be prevented by appropriate clinical measures (Hanlon et al., 2018). An optimal screening for FS in cardiovascular disease should be practical, sensitive, and approved for a specific patient population (Kim et al., 2016). In the literature, there are one-dimensional tools for assessing FS most often intended to screen for physical frailty, but multidimensional tools are becoming popular in clinical practice. The most frequently cited assessment of FS includes Frailty Phenotype, Frailty Index, and Clinical Frailty Scale (Khezrian et al., 2017). Multidimensional measures of FS can provide clinicians with more data on patients’ needs, their initial vulnerability, and also enable individualized therapeutic management. There is empirical evidence in support that FS is reversible. Thus, planned cardiac rehabilitation programs can help improve patients’ functional fitness, their ability to perform exercises, enhance psychosocial well-being, nutritional status, independence, and reduce the risk of death (Sepehri et al., 2014). Such multidimensional interventions of FS by focusing on several frailty components provide greater efficiency in the treatment and diagnosis of cardiological patients (Uchmanowicz et al., 2018).

The ICFSR guidelines include a recommendation for implementing comprehensive care with physical frailty that handles sarcopenia, treatable causes of weight loss, and the causes of exhaustion (depression, anemia, hypotension, hypothyroidism, and vitamin B12 deficiency) (Dent et al., 2019). To maximize benefits of frailty-reversing activities for patient with frailty, identification of its determinants appears to be fundamental. This multi-dimensional holistic approach is in favor of better diagnosis FS symptoms than the pure physical phenotype approach. The identification and further treatment of patients with cardiovascular disease based on the modified or reversed FS parameters directly translate into better treatment outcomes.

**FIGURE 1:**
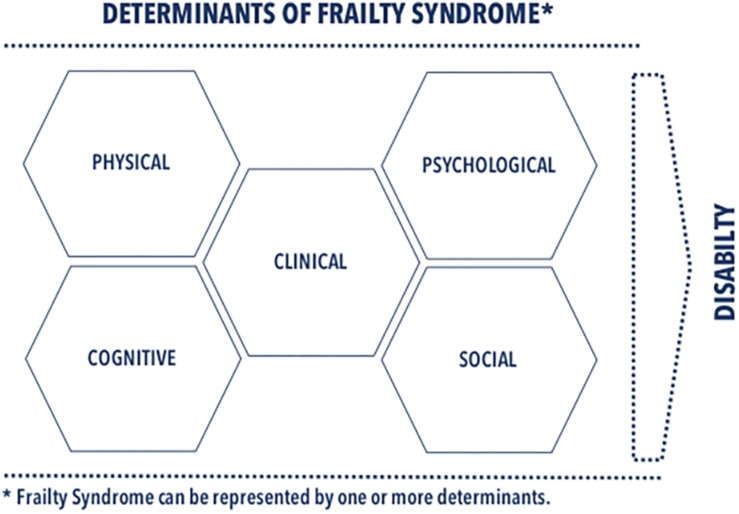
Determinants of FS.

The main goal of this review is to provide a detailed scrutiny of the frailty determinants presented in the recent literature on cardiology and cardiological nursing. We argue in this review for determinants, favoring a multidimensional assessment of FS in both research and clinical practice. As illustrated in Figure 1, we classified the determinants into several domains: clinical, physical, psychological, cognitive, and social ones. We complemented classification of each determinant with information necessary for its identification. This review emphasizes a multidimensional approach accommodating complexity of FS phenomena in research and clinical practice as a holistic approach to FS diagnosis and individualized therapeutic strategies that reduce the adverse effects of FS.

## Definition

The word frailty comes from the French language from the word *frêle*, which means: fragile, weak, delicate (Diaz et al., 2015). The notion of FS one usually understands as a syndrome of weakness, fragility, or exhaustion of reserves. In the past, FS was only a determinant of biological age until clinical observations that patients’ responses to the disease, their functional state, and survival depend not solely on the age factor, but by the physiological resources of the organism. Although there are many reports confirming that there is a relationship between FS and age, today’s view suggests FS to be not an unavoidable part of the aging process and does not apply only to the elderly. Therefore, FS goes beyond the physiological process of organism aging. For instance, FS affects younger patients with chronic diseases or cognitive dysfunctions (Bagshaw et al., 2014). In clinical medicine, there is still no common definition of FS, which is often referred to as a syndrome or condition.

One definition of FS states that it is: “*a physiological syndrome, characterized by a reduction in reserves and resistance to stressors, resulting from the accumulation of reduced performance of different physiological systems, which in turn leads to susceptibility to adverse consequences*” (Fried et al., 2001).

According to another definition, FS is: “*a multidimensional syndrome of homeostatic reserve loss (energy, physical and mental abilities), which promotes the accumulation of deficits, increasing the patient’s sensitivity and risk to adverse medical consequences*” (Clegg et al., 2013; Rajabali et al., 2016).

In the 2013 consensus of six geriatric societies assumed that FS is: “*a multi-causal clinical syndrome, characterized by a decrease in strength, endurance and reduction in physiological processes, increasing an individual’s susceptibility to development of dependency and/or death*” (Morley et al., 2013).

There are two dominant approaches to defining FS, a phenotypic definition of weakness and a definition based on the accumulation of deficits. Fried et al. (2001) proposed the first one based on data from the Cardiovascular Health Study. The second approach uses the frailty index from a Canadian study by Rockwood et al. Both approaches show a similar predictive accuracy in the identification of FS (Graham and Brown, 2017). Phenotypic frailty arises from age-related biological changes that shape the physical features of frailty (unintentional weight loss, weakening of muscle strength and mass, slower walking, reduced energy, decreased level of physical activity). The frailty model based on the accumulation of deficits recognizes that frailty results from an accumulation of abnormal, not only physical, clinical features including cognitive disorders, depressive symptoms, reduced functionality, multiple diseases, malnutrition, social isolation; their high accumulation speeds up the aging of the body. Phenotypically, physical features are a manifestation of frailty, whereas in terms of the accumulation of deficits are considered as a cause of frailty. The phenotypic approach is one-dimensional, and the one based on the accumulation of deficits is multidimensional (Robinson et al., 2013, 2015).

Multidimensional definitions are becoming increasingly important because FS results from negative effects of various factors on the body’s physiology, which increase its vulnerability to even potentially harmless stressors (Kovacs et al., 2017). Therefore, the definition of FS should consider not only the functional state but also the psychosocial weakness, and explicitly shows that a patient with concomitant FS is at risk of complications and susceptible to poor clinical outcomes (Robinson et al., 2013). The literature on FS also defines a pre-frail condition, which identifies individuals at risk of FS (Fried et al., 2001). Since frailty is a reversible state, several targeted interventions can prevent the transition from the pre-frail condition to fully symptomatic FS (Summers et al., 2018). The formulation of a single, common definition of FS appears to be important from both a scientific and clinical point of view. It will enable more accurate assessments of the prevalence of frailty in specific patient populations, facilitate comparisons of research findings and the better availability of meta-analytic scientific data. A single, common definition of FS in clinical practice would also help clinicians to select screening methods.

## Epidemiology

In the literature, there are various epidemiological data on the prevalence of FS, because of different research methods to identify it and the patient population assessed (Chen, 2015). Symptoms of FS occur in one in 10 elderly people (Goldfarb et al., 2015). Recent reports suggest that in Europe, 17% of elderly people have FS, while in Poland, the figure stands at 6.7%. In people over 80 years of age, the prevalence of FS in Poland increased up to 50% (Łęgosz et al., 2018). The meta-analysis and systematic review of studies of frailty in 22 European countries in the program of ADVANTAGE Joint Action showed that FS is widespread in Europe, and its actual prevalence varied across the studies and strictly depended on an operational definition of FS. For example, one study included in the analysis showed that the prevalence of FS in a patient population ≥80 years in the community is 7.2% (O’Caoimh et al., 2018). A recent study based on the phenotypic frailty model showed the prevalence of FS at 9.9%, while that of the pre-frail condition at 44% (Furukawa and Tanemoto, 2015). In a study on the accumulation of deficits in surgical patients, FS was in 28% of patients and the pre-frail condition in 20% of patients (Robinson et al., 2013). In a systematic review of 15 FS studies involving 44,894 patients, frailty was found in 9.9% of patients.

The prevalence of FS increased with age and was more common in women than in men (Oresanya et al., 2014). The Women’s Health and Aging study identified frailty in 11.3% of women. FS is more common in African-Americans and Asians than in Caucasians, single people and those with lower levels of education (Chen, 2015). FS patients are older, more often female, have more co-morbidities and a higher perioperative risk. In addition, they have a lower New York Heart Association (NYHA) class, poorer kidney function, higher NTPproBNP (N-terminal pro-brain natriuretic peptide), more depressive symptoms, higher frequency of mobility restrictions in basic and complex everyday activities, and poorer results in quality of life studies (Rodríguez-Pascual et al., 2016). After over 4 years of observation, out of 54.4% of elderly patients without FS, but almost half of the patients suffered from pre-frail status (Chen, 2015). Pre-frail status indicates a fourfold higher risk of developing FS within 4 years of observation (Sergi et al., 2015). In patients with cardiovascular diseases, the incidence of FS ranges from 10 to 60%, whereas in patients undergoing cardiac surgery in old age, it is even 50% (Graham and Brown, 2017; Zuckerman et al., 2017). In the Frailty Assessment Before Cardiac Surgery (ABCS), 46% of patients aged 70 years or older undergoing coronary artery bypass and/or heart valve surgery were frail in a 5-m gait rate test (Afilalo, 2011). A recent report suggests that preventing FS could delay 2–5% of deaths (Łęgosz et al., 2018).

## Pathophysiology

Frailty syndrome pathophysiology arises primarily from a metabolic imbalance of the body and impaired functioning of the immune and endocrine systems. There is a hypothesis that combined processes of apoptosis, aging, autophagy, and mitochondrial dysfunction play a key role at the cellular and molecular levels. Disturbed cellular processes influence the development of FS through changes in the functioning of organs and systems (Graham and Brown, 2017). The changes in FS resemble and often overlap with the physiological aging process, but in FS they are mainly concentrated on a disturbed energy metabolism, which is the imbalance between the anabolic state and the catabolic state. Thus, frailty is often associated with metabolic deficiencies, increased nutritional risk, and sarcopenia, which is defined as a decrease in muscle mass, strength, and capacity (Joyce, 2016; Cruz-Jentoft et al., 2019).

In cardiovascular diseases, inflammation plays a key role in lipoprotein oxidation and platelet activation. Chronic inflammation in FS induces catabolism, which results in a redistribution of amino acids from skeletal muscles, leading to a deep loss of muscle mass. As muscles are the main reservoir of amino acids, losing muscle mass and change in their metabolism impair the body’s ability to repair itself when confronted with stressors. Hence, muscle mass loss is an essential component of FS (Afilalo, 2011). The presence of chronic diseases, such as heart failure, and surgical procedures additionally contribute to the stimulation of the immune and sympathetic systems, causing inflammation manifested by high levels of C-reactive protein (CRP), elevated white blood cell count, and interleukin 6 (IL-6) (Soysal et al., 2016). Pro-inflammatory cytokines may affect frailty either directly, promoting protein degradation, or indirectly affecting important metabolic pathways. In their meta-analysis of 32 cross-sectional studies (23,910 elderly people), Soysal et al. (2016) observed that frailty and pre-frail status were associated with a significant increase in serum inflammatory factors, in particular with a high increase in CRP and IL-6. In patients with frailty and pre-frail status, disability and obesity were more frequent as coexisting factors increasing inflammatory parameters. Individuals with coexisting FS are characterized by weakened immune system, reduced T-cell activity, and antibody production, and an increase in oxidative stress products, which ultimately leads to increased inflammatory parameters in the blood serum. Apart from CRP and IL-6, patients with FS experience an increase in tumor necrosis factor (TNFα), fibrinogen and D-dimers, low vitamin D concentration, decreased concentration of sex hormones and growth hormone, abnormal secretion of cortisol, or high level of C-glycosyl tryptophan (Życzkowska and Grądalski, 2010; Soysal et al., 2016; Koh and Hwang, 2019).

## Determinants

### Reduction in Physical Activity

The decrease in physical activity, which is one of the determinants of FS in combination with the coexistence of chronic diseases, contributes to the acceleration of catabolic processes and consequently leads to disability. In cardiac surgery, a decrease in functional efficiency is observed in 16% of elderly patients and 20% of patients aged ≥70 years (Hoogerduijn et al., 2014). Decreased functional efficiency in cardiac patients often results in a loss of autonomy, increased dependence on others, and reduced quality of life. Moreover, it is associated with longer hospital stays, increased use of health care resources, institutionalization, and mortality (Hoogerduijn et al., 2014). In a randomized surgical treatment for ischemic heart failure (STICH) study, patients qualified for CABG with improved functional performance showed a lower perioperative risk and lower mortality during 5 years of follow-up (Singh et al., 2014). Patient mobility as one of the components of FS, assessed by the walking speed test, is a recognized, sensitive indicator and predictor of institutionalization, disability, and mortality after cardiac surgeries (Gobbens and van Assen, 2014; Kim et al., 2016). In patients with reduced walking speed and high perioperative risk, the incidence of mortality was 43% compared to patients with normal gait rate and medium and low perioperative risk, where it was 6%. Meta-analytic data based on nine prospective studies showed that an improvement in gait rate by 0.1 m/s leads to a 10% improvement in survival (Afilalo et al., 2014). Patients’ dependence with respect to basic vital functions or the use of auxiliary devices are independent predictors of test results after cardiac surgeries (Neupane et al., 2017).

The walking speed test also has a positive prognostic value in predicting disability in the areas of activities of daily living (ADL) and instrumental ADL (IADL) (Gobbens and van Assen, 2014). Hospitalization often leads to the impairment of functional performance and development in one-third of patients with disabilities, especially with problems with early activation of patients after medical procedures. When activating patients after cardiac surgery for an average of 43 min a day, there is a risk of losing 1–5% of muscle strength every day, which significantly increases the risk of developing disability, especially in patients with concomitant FS (O’Neill et al., 2016). The gait speed is a clinical marker of physical frailty, often used in cardiac surgery for predicting the risk of perioperative complications in elderly patients. A cut-off for slow gait speed is present in a walk slowdown on a distance of 5 m in ≥6 s (walking speed of ≥0.83 m/s) (Afilalo et al., 2010).

### Malnutrition

Abnormal nutrition status of the patient plays an important role among FS determinants. Malnutrition contributes to the reduction of muscle mass and strength, thus impairing the physical performance of the body. Moreover, it increases the dysfunction of the immune system, thus reducing the resistance to infection. In general, it seems that anorexia related to aging and the associated weight loss play an important role in the pathophysiology of frailty (Fougère and Morley, 2017). Weight loss in elderly people is most often unintentional (Gaulton and Neuman, 2018). According to the phenotypic approach, frailty is determined by unintended weight loss of more than 4.5 kg or ≥5% over the last year (Fried et al., 2001).

Depending on the tool used to assess the nutritional status, the percentage of malnourished patients before cardiac surgery varies between 4.6 and 19.1% (Lomivorotov et al., 2013). In patients qualified for cardiac surgery, abnormal nutrition correlates with increased morbidity, mortality, prolonged hospitalization, abnormal wound healing, and delayed benefits of postoperative cardiac rehabilitation (Arai et al., 2018; Jayaraman et al., 2018). Pre-operative identification of nutritional risk is extremely important for predicting complications and surgical results in cardiac surgery (Ringaitiene et al., 2016). Unfortunately, nutritional risk often remains undiagnosed in cardiac patients, and thus inadequately treated. Studies confirm that patients undergoing cardiac surgery are at a greater risk of iatrogenic malnutrition due to discontinuation of food supply in the early postoperative period (Hill et al., 2018). Most patients are admitted to cardiac surgery from 12–24 h prior to the procedure, which makes it impossible to undertake appropriate nutritional interventions even though the nutritional status has been assessed. Nutritional status assessed before cardiac surgery would provide an early opportunity to implement nutritional interventions and optimize the nutritional status of the patient before surgery. Studies have shown that obese patients have a higher incidence of complications after cardiac surgeries than those with normal body weight or overweight, but have lower short-term mortality rates (Gaulton and Neuman, 2018). Mini Nutritional Assessment-Short (MNA-SF) is a recommended tool for the identification of malnutrition in elderly cardiac patients (Goldfarb et al., 2018).

### Sarcopenia

Sarcopenia is the biological basis of the frailty phenotype. The name sarcopenia derives from the Greek language from the words “sarx,” meaning body, and “penia” meaning loss. Sarcopenia does not occur in every patient with FS (Morley, 2016). However, the overlap between sarcopenia and frailty ranges from 50 to 70% (Morley et al., 2014). The pathophysiology of sarcopenia is multifactorial and includes, among others, mitochondrial dysfunction, loss of motor neurons, inadequate nutrition, poor absorption, increase in inflammatory cytokines, insulin resistance, growth hormone deficiency, or androgen deficiency. The decrease in physical activity is very important in the pathophysiology of sarcopenia (Morley, 2016).

Sarcopenia is defined as age-related loss of muscle mass and strength. Studies have shown that every year people lose 1–2% of their skeletal muscle mass and the muscle strength is reduced by about 3–4%. This loss is accelerated in patients with FS. If an additional stress factor, i.e., cardiac surgery, is triggered, the patient with sarcopenia has a problem with protein compensation in the amount necessary for proper wound healing or immune system functioning. The demand for protein in such a patient increases even up to 400%. Combination of anabolic insufficiency and stress factors accelerating catabolism is further aggravated by immobilization of the patient in bed or malnutrition, which induce rapid muscle loss and the occurrence of complications. In the case of a patient with FS, even a slight loss of 5% of muscle mass may cause adverse health effects (Afilalo, 2016).

Sarcopenic obesity refers to a subgroup of people with sarcopenia and a high fat content. In addition to low lean body weight or low muscle capacity, the disease is characterized by excessive energy intake, low physical activity, low intensity inflammation, and insulin resistance. This is a subgroup which for some time has been attributed a high risk of complications (Rizzoli et al., 2013). With age, the lean body mass decreases and is replaced by fatty tissue, whose distribution changes. The amount of subcutaneous fat decreases, while that of visceral fat increases. This happens regardless of the classical body mass index (BMI). Therefore, its use may be inadequate among the elderly, in whom an increase in fat mass and a decrease in lean body mass contribute to ill health (Ricci et al., 2014; Badrudin et al., 2016).

The European Working Group on Sarcopenia in older people (EECSOP) recommends administration of the SARC-F questionnaire for screening sarcopenia. To assess muscle strength, one recommends a grip strength or chair stand test (chair rise test). For assessing skeletal muscle mass and quality consensus recommends tests such as dual-energy X-ray absorptiometry (DXA), bioelectrical impedance analysis (BIA), computed tomography (CT), or magnetic resonance imaging (MRI). Whereas, in terms of physical performance, the recommended measurements include a walking speed test, short physical performance battery (SPPB), timed-up-and-go test (TUG), 400-m walk, or long-distance corridor walk (400-m walk) (Cruz-Jentoft et al., 2019).

### Polypharmacy

Polypharmacy is a common and potentially modifiable risk factor for frailty in elderly people. Polypharmacy, defined as the use of at least five drugs simultaneously, increases the risk of mistakes in drug dosing by the elderly and the occurrence of adverse reactions. Age-related changes in pharmacokinetics and pharmacodynamics of drugs, as well as multi-morbidity, make prescribing drugs for the elderly a clinical challenge (Saum et al., 2017). Polypharmacy is associated with an increased risk of frailty during 8 years of observation, even after taking into account multi-morbidity. The risk of FS increased by 55% in patients treated with four to six drugs and 2.5 times in patients treated with more than seven drugs. Veronese et al. (2017) observed that the inclusion of each additional drug was associated with an 11% increase in the risk of frailty. Another study on polypharmacy showed that it increases 1.5–2 times risk of frailty development within 3 years, regardless of the number of concomitant diseases and their severity (Saum et al., 2017). Polypharmacy may contribute to the development of frailty through negative effects on coexisting diseases and additional factors (e.g., weight loss) stated in the definition of frailty. Polypharmacy-related side effects may further increase the risk of FS as they often lead to the so-called prescribing “cascade,” in which new drugs are prescribed to counteract adverse effects of drugs taken so far (Veronese et al., 2017).

In elderly patients, multi-morbidity is common, and this group is particularly susceptible to polypharmacy. Multimorbidity is a factor driving polypharmacy and conducive to the development of FS (Payne, 2016; Yarnall et al., 2017). The overlap of these two concepts is clear and most research investigates this area in parallel, not in cooperation. As noted by Sinnott and Bradley (2015), multi-morbidity and polypharmacy may coexist, hence the recognition of both concepts as FS determinants seems to be present in many studies. Nevertheless, close monitoring for polypharmacy should be advised to assure better clinical outcomes in frail patients (Bonaga et al., 2018). It is necessary to conduct further studies to verify whether the reduction of polypharmacy has a positive effect by modifying, limiting, or delaying FS (Gutiérrez-Valencia et al., 2018).

### Depressive Symptoms

Depression is one of the main determinants of frailty in elderly people (Joyce, 2016). It has been found that the prevalence of FS in people with depression is 40.4%. Depression increases the risk of FS four times, and frail individuals are more likely to develop depression. This means that the presence of frailty poses a risk of developing depression and the presence of depression poses a risk of developing frailty (Soysal et al., 2017). These two constructs overlap. Symptoms indicating depression may be difficult to identify clinically due to the coexistence of frailty in old age. Symptoms such as decreased daily life activity may be the result of reduced energy reserves, characteristic of frailty but also of anhedonia depression, or the result of disability, which causes loss of ability in this area. However, a meta-analysis by Vaughan et al. (2015) indicates a stronger relationship between depressive symptomatology and increased risk of frailty. The literature also describes the relationship between antidepressant treatment and increased incidence of frailty in elderly women.

The coexistence of depression and frailty in the elderly has several pathophysiological mechanisms. One of such overlapping mechanisms is subclinical cerebral vascular disease, which assumes that mood changes and cognitive disorders in the elderly are caused by subclinical cerebral vascular ischemia. More and more evidence also confirm the role of chronic inflammation as a causative mechanism of both depression and frailty in elderly people. Similarly, an “inflammatory hypothesis” has been proposed for geriatric depression, in which inflammatory processes are believed to cause changes in the nervous system, which predispose some patients to the development of geriatric depression. Among pro-inflammatory cytokines, elevated levels of IL-6 were consistently associated with significant depressive symptoms in elderly people. Other possible etiological factors of both depression and FS in the elderly include disorders of hypothalamic-pituitary-suprarenal regulation, age-related testosterone reduction, or daily fluctuations of cortisol (Vaughan et al., 2015).

Anxiety and depressive symptoms are associated with cardiovascular incidents. In cardiac patients, subjective evaluation of patient anxiety was associated with a higher risk of mortality and in-hospital morbidity, taking into account perioperative risk and symptoms of preoperative depression measured with the hospital anxiety and depression scale (HADS-M). Generalized anxiety disorders are associated with perioperative complications in the form of serious cardiovascular incidents (MACCEs) after CABG surgery (Tully et al., 2015).

Since the occurrence of depressive symptoms and the level of anxiety are potentially modifiable, identification of these factors may provide a chance to increase mental comfort and improve clinical outcomes (Williams et al., 2013). Since depression is a psychiatric determinant of FS, one should also mention the other relevant neuropsychiatric symptoms of apathy common in the elderly population. Apathy symptoms more likely result from damage to the fronto-subcortical pathways that manifest in declining cognitive, emotional, and motoric goal-directed behavior (Ayers et al., 2017). Although apathy in displayed symptoms resembles depression, clinically this other pronounced psychiatric condition that can occur in the absence of depression and apathy pose a certain diagnostic challenge. In fact, clinical studies show some correlations between apathy and depression based on the rating scales, although careful quantification of these measures challenges similar symptomatology of both disorders. The findings from neuroimaging support the notion that apathy is not depression as neuropathology specific for both conditions involve different brain regions. In old age, apathy may become a more significant feature of depression, so it is greater in in late-onset depression than in early-onset depression (Ishii et al., 2009). In the study of Ayers et al. people with initial apathy had more than twice the risk of slowing down gait and over three times the risk of disability, which shows the general risk of a decrease in functional efficiency associated with apathy in the elderly. This risk increases with the increase in apathy. This relationship was independent of depressive symptoms even after taking into account demographic factors, health status and cognitive functioning (Ayers et al., 2017).

### Cognitive Disorders

Cognitive disorders are considered by some researchers to be one of the predictors of FS (Uchmanowicz et al., 2015a). FS may be treated as an indicator of future cognitive disturbances (Uchmanowicz et al., 2015a). Clinical data suggest a clear relationship between FS and mild cognitive impairments, dementia, cognitive decline in late age, and dementia without Alzheimer’s disease in the elderly. A recent systematic review along with the meta-analysis showed a relationship between FS of the elderly and the risk of developing cognitive impairment, especially components of frailty were related to vascular dementia in patients with cardiovascular disease (Borges et al., 2019). In the elderly, frailty is associated with lower global or regional brain volume, a higher number of cerebral microbleeds, and a higher burden of white matter hyperintensities (WMHs) of presumed vascular origin. The study by Kant et al. investigated brain damage in individuals with frailty and found reduced total brain volume and gray matter volume in these patients as opposed to pre-frail and non-frail populations. In addition, individuals with physical frailty and those classified as pre-frail displayed more cerebral infarctions as compared to individuals without frailty. The authors suggested that plausibly the phenotype of physical frailty originated these brain abnormalities (Kant et al., 2018).

Cognitive functions include a range of intellectual processes such as short-term memory, long-term memory, writing, reading, speech, visual and spatial processes, abstract thinking, and the perception of external stimuli. When fully maintained, cognitive abilities enable biopsychosocial functioning on a daily basis. Physiologically, aging processes include age-related memory impairment or age-related cognitive decline (Ishizaki et al., 2006). The International consensus group has identified the coexistence of physical frailty and cognitive deficits in the elderly as cognitive frailty (Uchmanowicz et al., 2018). Patients with cognitive frailty are at a greater risk of disability, limited daily functioning and hospitalization. Pro-inflammatory cytokines play an important role in the pathophysiology of both conditions, and WMH is associated with both cognitive impairment, decreased walking speed, and risk of falls (Morley, 2016). The notion of cognitive frailty describes what is an individual’s reduced cognitive reserve which is potentially reversible as opposed to physiological brain aging (Facal et al., 2019).

There are studies on cognitive decline in patients undergoing cardiac surgery, which substantially increases the risk of cognitive decline after surgery and the occurrence of postoperative delirium. Postoperative decline in cognitive function is more frequent in patients with pre-existing cognitive disorders (Neupane et al., 2017). There is a correlation between cognitive impairment and higher dependence regarding basic vital functions within 6 months after cardiac surgery (Lindman and Patel, 2016). There are common tools for identifying cognitive impairment in patients with FS such as the Mini-Mental State Examination (MMSE). This is a short easy-to-use questionnaire, suitable to screen for impairment in cognitive function of orientation in time and place, remembering, attention and counting, recalling, language functions, repetition, construction praxis (Hao et al., 2018).

### Lack of Social Support

According to the English Longitudinal Study of Aging (*ELSA study*), social isolation and loneliness have turned out to be independent factors of FS and have been associated with old age, a lower level of education, a lower economic status, the occurrence of depressive symptoms, a greater number of chronic diseases, and more frailty criteria met. In this study, social isolation has been associated with an increased risk of the pre-frail condition. Loneliness is an important predictor of physical frailty progression, and FS is associated with a greater likelihood of loneliness, which shows a two-way relationship between them. Both social isolation and loneliness are associated with an increase in mortality, an increased risk of cardiovascular incidents, and a decrease in functional performance. Both social isolation and loneliness are associated with a decrease in gait speed (Gale et al., 2018). Recovery after cardiac surgery is largely based on the patient’s social structure, and unfavorable health behaviors contribute to increased morbidity and mortality in cardiac surgery patients (Synowiec-Piłat et al., 2014). There are social factors which increase the perioperative risk by making the patient susceptible. These factors include: the lack of social support, loneliness, a remote place of residence, difficult access to healthcare, a low socioeconomic status, and a lower level of education. What is important is that these factors appear to be independent of the biological and physical stress associated with cardiac surgery (Neupane et al., 2017).

The Tilburg frailty indicator is a multidimensional tool for assessing FS and allows to get data on frailty in social domain (Gobbens et al., 2010b). Another tool for assessing social support administered to patients with chronic diseases is a multidimensional scale of perceived social support (MSPSS) (De Maria et al., 2018).

## Frailty, Multi-Morbidity, Disability

Frailty syndrome, multi-morbidity, and disabilities are closely linked but separate constructs. Multi-morbidity is defined as the presence of two or more diagnosed chronic diseases in a given patient, constituting a measure of their individual state of health. Disability, on the other hand, is defined as functional problems in the performance of everyday activities necessary for independent living and reflects the interaction between the individual and the surrounding environment. Therefore, multi-morbidity should be understood as one of the main causes of FS, and disability as one of its negative consequences. Disability is the final stage, a side effect of FS and human environmental stressors (Afilalo, 2016). FS may precede or coexist with disability (Robinson et al., 2015).

Multi-morbidity occurs in 16% of patients over 65 years of age and 35% of patients over 80 years of age. Multi-morbidity has a key influence on the diagnostic and therapeutic process, because the manifestation of disease symptoms may differ and make their interpretation difficult. Multi-morbidity is associated with a higher risk of death, higher rate of rehabilitation, disability, and reduced quality of life (Pulignano et al., 2017). Optimization of the clinical status of multi-morbidity patients seems to be important in the context of the perioperative risk in cardiac patients.

Disability is most often determined by difficulties in basic daily activities (ADL) and/or complex daily activities (IADL). The Katz scale (ADL) and the Lawton scale (IADL) are the most common tools used in the literature to determine disability. The ADL includes activities such as bathing, dressing and undressing, using the toilet, getting up from bed and moving to a chair, eating, and controlling the excretion of urine and bowel movements. The IADL includes activities such as using the telephone, walking, shopping, preparing meals, do-it-yourself activities, doing laundry, preparing and taking medication, and managing money. Difficulty in performing both basic and complex everyday activities means total disability (Chen, 2015). Disability also occurs in patients qualified for cardiac surgery (Afilalo et al., 2012; Lindman and Patel, 2016). In their study, which concerned the inclusion of disability, among other factors, in the assessment of perioperative risk in cardiac patients, Affilalo et al. observed disability in 5% of patients with respect to basic vital functions and in 32% of patients with respect to complex vital functions. The authors of this study propose a Nagi scale for the evaluation of disability in cardiac surgery, which seems to be more sensitive in its diagnosis and in this case affected 76% of patients (Afilalo et al., 2012). In another study on cardiac patients, Sun et al. (2018) found that disability is more common than mortality 1 year after surgery, and that the risk factors for disability are female gender and heart failure. Given the impact of disability on the quality of life of elderly people, frailty gains in importance. It can represent the intervention-prone condition prior to disability and identify surgical patients with a high probability of developing disability (Graham and Brown, 2017).

## Discussion

Our review provides the multidisciplinary approach to understanding measures of FS in cardiological populations. In today’s clinical practice in cardiovascular diseases, none of the multivariate measurements of FS is practically available for clinicians. Here, we show that clinician knowledge should take into account several important determinants of frailty that pose risk factors of the negative course of the disease and its adverse health consequences for patients. The frailty determinants in this work are in line with the views presented in the recent literature, emphasizing the combined effect of several determinants on FS in a cardiac patient. For example, the article by Vitale et al. (2018) defines overlapping frailty that includes several domains such as cognitive deficits, functional impairment, physical deficits, mood disorders, undernutrition, or no social support. These accumulating deficits driven by FS determinants contribute to decreasing resources in stress resistance as showed in the recent literature. As indicated by Vitale et al. (2018), although this multidisciplinary approach should be a part of a holistic therapeutic plan to treat frail patients, there are still no relevant standards in clinical practice. In fact, clinicians based the FS rating for a long time solely on the physical dimension of frailty.

The multifaceted dimension of FS departs from the purely physical definition and emphasizes the possibility of deterioration in many areas of functioning (McDonagh et al., 2018). Uchmanowicz et al. (2015b, 2019) argue that adverse outcomes of frailty are patient rehospitalization, level of self-care, mortality, patient morbidity, and deterioration of patients’ quality of life. For instance, van der Vorst et al. (2018) showed that frail older adults from the multidimensional perspective are likely at the greater risk of dependency in ADL. Thus, as Gobbens et al. (2010a) proposed, physical, psychological, social losses in several domains of human functioning are better predicted by the integral, definition of frailty which is “a dynamic state affecting an individual who experiences losses in one or more domains of human functioning ([…]) that are caused by the influence of a range of variables and which increases the risk of adverse outcomes.” The position paper of Vitale et al. (2019) based on Heart Failure Association experts stress a holistic approach to frailty as more credible than a simplistic, physical approach of FS showing in this fashion that the nature of frailty is dynamic and multidisciplinary, and not influenced by the age factor. Following this account on FS, Vitale et al. (2019) propose Heart Failure Association Frailty Score scale, the rapid and easy-to-use measurement to evaluate four clinical, psycho-cognitive, functional, social in frail patients.

To sum up, understanding frailty and its determinants seems to be crucial for the diagnostic and therapeutic process for cardiology, ultimately leading to targeted interventions with a better potential to reverse the effects of frailty and prevent further complications in cardiac patients. In this review, we attempted to identify the essential determinants of FS based on the multidisciplinary approach. Here, we argue that this way of tackling FS is necessary if one wants to assess frail patients on individual determinants. However, we mainly focus on the significance of individual determinants frailty and therefore other important aspects of FS linked with interventions may be at some point neglected in the presented review. Nevertheless, future research on FS should seek a multidisciplinary definition of frailty embracing wider populations with cardiovascular diseases in order to adopt efficient measurements of FS, building targeted, fragility-reversing therapeutic strategies and guidelines into everyday clinical practice.

## Summary

This review attempted to identify the critical determinants of FS embracing this complex medical syndrome from a multidimensional perspective and cardiological conditions. We analyzed individual determinant and added concrete proposals of tools for their FS identification. Undoubtedly, a challenge for modern cardiology both in the stream of future research and in everyday clinical practice is to build a clear definition of frailty. It seems that the adoption of a multidimensional definition is promising, because it ends up with the practical tool in designing strategies and interventions to prevent the development of frailty. Knowledge of individual FS determinants is important for clinicians in identifying individual patient’s needs, adapting to them therapeutic strategies, risk stratification, clinical decisions-making, and building programs that would reverse symptoms of FS and reduce the medical, psychological, social, and economic costs incurred for the adverse consequences of FS.

## Conclusion

Frailty syndrome is a reversible clinical condition. For planning and implementing appropriate measures to prevent the occurrence of FS or minimize its negative health consequences for cardiological patients, important are comprehensive definitions of FS, familiarity with the prevalence of FS in a variety of patient populations, in-depth knowledge of pathophysiology, and additional factors of multi-morbidity and disability in frail patients. The multidimensional approach toward FS adapts individualized interventions for a single patient with cardiovascular disease. Our review shows that insight into FS determinants is the starting point for building both the comprehensive definition of FS and the adoption of the assessment method of FS, and then successful clinical management.

## Limitations

This review mainly refers to frailty determinants in cardiovascular diseases. In this article, we provide neither references on other chronic diseases nor discussion of identifying frailty determinants in individuals without diagnosed chronic diseases. In addition, because of the limited volume, this article scrutinized only tools for identifying individual FS determinants and abandoned their relevant detailed descriptions. The review did not discuss specific strategies for individual determinants to get them clinically reduced for a patient. However, this will be the subject of a future publication, continuing this topic.

## Author Contributions

All authors listed have made a substantial, direct and intellectual contribution to the work, and approved it for publication.

## Conflict of Interest

The authors declare that the research was conducted in the absence of any commercial or financial relationships that could be construed as a potential conflict of interest.
